# Ascending colon cancer accompanied by tumor thrombosis in the superior mesenteric vein: A case report

**DOI:** 10.1016/j.ijscr.2020.07.018

**Published:** 2020-07-17

**Authors:** Yoshiaki Fujii, Kenji Kobayashi, Sho Kimura, Shuhei Uehara, Shuji Takiguchi

**Affiliations:** a Department of Surgery, Kariya Toyota General Hospital, Aichi, Japan; b Department of Gastroenterological Surgery, Nagoya City University Graduate School of Medical Sciences, Aichi, Japan

**Keywords:** SMV, Superior mesenteric vein, IMV, Inferior mesenteric vein, CT, Computed tomography, ICV, Ileocolic vein, ICG, Indocyanine green, NIR, Near-infrared, Colorectal cancer, Superior mesenteric vein, Tumor thrombosis, Case report

## Abstract

**Introduction:**

Venous tumor thrombosis occasionally accompanies renal cancer, liver cancer, and pancreatic cancer. Colorectal cancer is seldom accompanied by venous tumor thrombosis in the portal vein or the superior or inferior mesenteric veins (SMV, IMV), and little is known about its features. We report a case of ascending colon cancer with tumor thrombosis in the SMV treated with right hemicolectomy and combined resection of the SMV.

**Presentation of case:**

An 82-year-old man with chief complaints of loss of appetite was admitted to our hospital. He was diagnosed with ascending colon cancer accompanied with tumor thrombosis extending to the SMV. He underwent right hemicolectomy and combined resection of the tumor thrombosis and the SMV. Intestinal blood flow was evaluated by intraoperative indocyanine green (ICG) fluorography. He continued to recover well from surgery. No adjuvant chemotherapy was employed because of the patient’s advanced age and his own will. He was transferred to another hospital on postoperative day 39. Six months after surgery, abdominal CT showed multiple liver metastases. He died 8 months after surgery.

**Discussion and conclusions:**

Radical resection of the primary tumor and surgical thrombectomy should be considered for the treatment of colorectal cancer without distant metastasis accompanied by tumor thrombosis. However, venous tumor thrombosis is representative of an aggressive cancer and that may be a strong risk factor for the development of liver metastasis. Adjuvant systematic chemotherapy in addition to complete surgical resection may be one of the treatment strategies.

## Highlights

• Colorectal cancer is seldom accompanied by venous tumor thrombosis in the superior mesenteric vein and little is known about its features.
• Venous tumor thrombosis is representative of an aggressive cancer and that may be a strong risk factor for the development of liver metastasis.
• Radical resection of the primary cancer combined with venous and adjuvant chemotherapy may be one of the treatment strategies.

## 1 Introduction

Renal cancer, liver cancer, and pancreatic cancer are occasionally accompanied by venous tumor thrombosis [1]. Colorectal cancer accompanied by tumor thrombosis in the portal vein, SMV or IMV is quite unfrequent. Otani et al. reported that venous tumor thrombosis may be a strong risk factor for liver metastasis and that the prognosis of patients may be poor [2]. However, following complete surgical resection with systematic chemotherapy, some patients can achieve a long survival. Radical resection of the primary tumor and surgical thrombectomy should be considered for the treatment of colorectal cancer without distant metastasis accompanied by tumor thrombosis. The surgical procedure of thrombectomy should provide oncological safety and avoid blood flow disorders. We report a case of tumor thrombosis in the SMV treated with right hemicolectomy and combined resection of the SMV. This work has been reported in line with the SCARE criteria [3].

## 2 Presentation of case

**Fig. 1:**
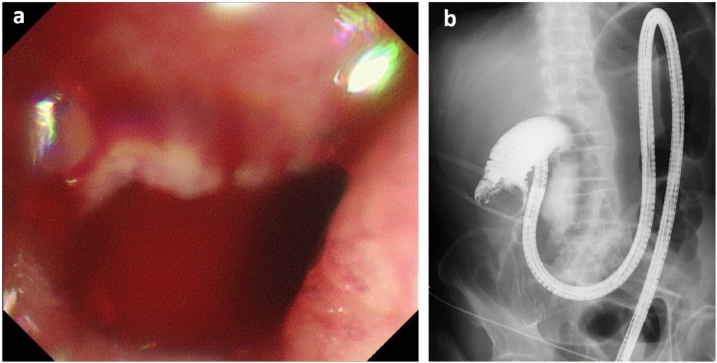
a) Colonoscopy revealed a type 2 tumor and circular stenosis in the ascending colon. The colonoscopy was unable to pass the lesion. b) Gastrografin enema revealed stenosis observed in the ascending colon.

**Fig. 2:**
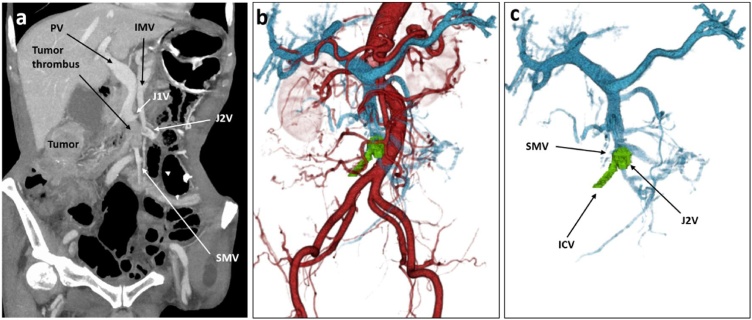
a) Dynamic CT revealed an intraluminal filling defect extending from the ICV to the SMV. There was no blood flow in the SMV with the tumor thrombus. b.c) On 3D-CT-reconstructed vascular images, the green lesion is the tumor thrombus. The shape of the tumor thrombus extended from the ICV to the SMV over a distance of 3.3 cm, including the second jejunal vein.

**Fig. 3:**
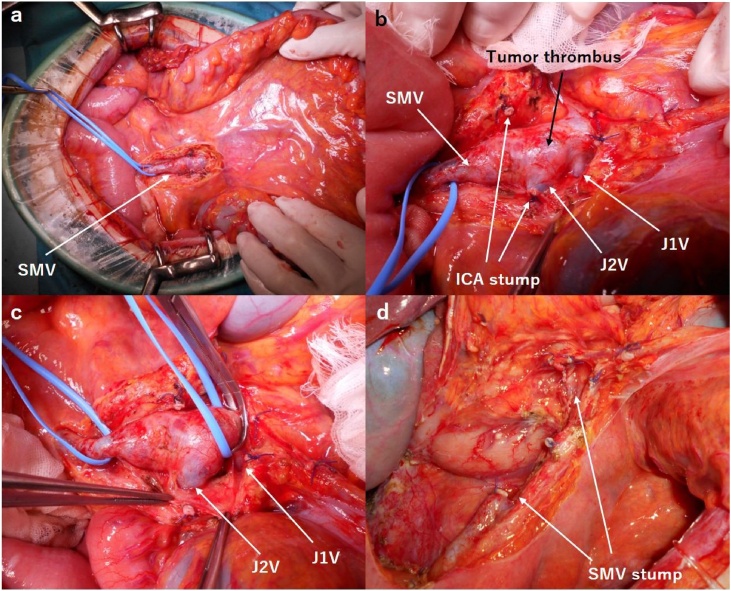
Intraoperative findings. a,b) To avoid the movement of cancer fragments during surgical resection, initial ligation of the SMV with the tumor thrombus is ideal. c) Even after clamping the SMV at the proximal end of the tumor thrombosis for 30 min, the small intestine showed no ischemic or congestive changes. d) We ligated the proximal and distal sides of the SMV with the tumor thrombus.

**Fig. 4:**
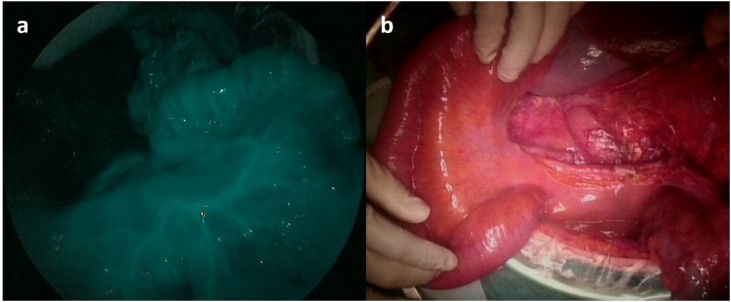
a) We confirmed good perfusion of intestinal artery blood flow using the NIR camera system after ICG injection. b) The small intestine showed no ischemic or congestive changes under the direct visualization. The macroscopic intestinal finding was normal during the almost 8 h operation.

An 82-year-old man presented to our hospital with complaints of fever elevation and loss of appetite. He had a medical history of Parkinson's disease, hypertension and hyperlipidemia. His laboratory results were as follows (Table 1). The tumor marker such as carcinoembryonic antigen and cancer antigen 19-9 was not measured on admission. Total colonoscopy revealed a protruding mass and circular stenosis in the ascending colon that almost obstructed the lumen (Fig. 1). The colonoscopy was unable to pass the lesion and moderately differentiated adenocarcinoma was diagnosed by examining the biopsy specimen. Dynamic CT revealed an enhanced mass in the ascending colon, a right iliopsoas abscess near the mass and an intraluminal filling defect extending from the ICV to the SMV (Fig. 2a). There was no swollen lymph node and no distant metastasis. The patient was diagnosed with ascending colon cancer with a right iliopsoas abscess and tumor thrombosis in the SMV. After admission, the inflammatory response improved with CT-guided drainage of the right iliopsoas abscess and antibiotic infusion. The patient underwent right hemicolectomy with D3 lymphadenectomy and combined resection of the SMV (Fig. 3). The tumor thrombosis extended from the ICV to the root of the SMV. We clamped the SMV above the proximal side of the tumor thrombosis for 30 min and used intraoperative ICG fluorography for evaluation of the intestinal blood flow (Fig. 4). After resection of the tumor combined with the SMV, the ileum and transverse colon were side-to-side anastomosed. The macroscopic intestinal finding was normal during the operation. The entire operative time was 461 min. The intraoperative blood loss was 985 mL.

**Table 1:** The laboratory results.

White blood cell	9.8 × 10^3^/μL
Hemoglobin	9.2 g/dL
Platelet	53.4 × 10^3^/μL
AST/ALT	17/11 IU/L
Plasma sodium	135 mEq/L
Plasma potassium	4.3 mEq/L
Plasma chloride	99 mEq/L
A blood urea nitrogen	18.7 mg/dL
Creatinine	0.51 mg/dL
C-reactive protein	16.2 mg/dL

**Fig. 5:**
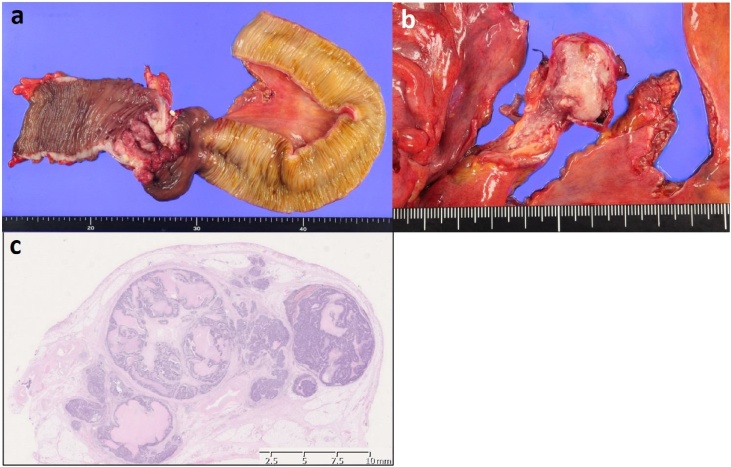
a) Pathological ﬁndings. There was a type 2 tumor of the ascending colon. b) White-colored tumor thrombus continuously filling from the ICV to the SMV. Histological findings revealed a tumor thrombus in the SMV. c) Tumor thrombus consisting of metastatic adenocarcinoma cells in the background.

Pathological examination showed that the tumor was a moderately differentiated adenocarcinoma of the ascending colon, reached the serosal layer, had 4 lymph node metastases (4/14), and lymphovascular invasion was observed. The resected venous portion from the ICV to the SMV was filled with white tumor thrombus (Fig. 5a, b). The tumor thrombus consisted of metastatic adenocarcinoma cells in the background (Fig. 5c). The circumferential resection margin was negative. The pathological staging was T4aN2aM0, stage IIIC (TNM classification).

He continued to recover well from surgery. Oral intake started on postoperative day 3 and was sufficient on day 7. However, his activities of daily living had weakened due to the preoperative hospital stay. So, he and his family hoped to transfer in order to take a physical therapy. The patient was transferred to another hospital on postoperative day 39. No adjuvant chemotherapy was employed because of the patient’s advanced age and his own will. He received a follow-up examination. Six months after surgery, abdominal CT showed multiple liver metastases and the tumor marker levels were increased: carcinoembryonic antigen, 5.2 ng/mL; cancer antigen 19-9, 7 U/mL. Palliative care was provided for the patient and he died of aspiration pneumonia 8 months after surgery.

## 3 Discussion

Tumor thrombosis in the portal vein, IMV or SMV originating from colorectal cancer is quite unfrequent. Tumor thrombosis originates from the primary lesion, liver metastasis, or extrinsic venous occlusion produced by enlarged metastatic lymph nodes [4]. In this case, the metastatic route of the intraluminal tumor thrombus originated directly from the primary lesion. Venous tumor thrombus was detected in 1.7% of patients with advanced colorectal cancer [5]. Otani et al. reported that 19.5% of venous tumor thrombosis from colorectal cancer was accompanied by synchronous liver metastasis [2]. Venous tumor thrombosis may be originated from an aggressive cancer and a strong risk factor for liver metastasis.

Dynamic CT is essential for the diagnosis and identifying the range of a venous tumor thrombus. The SUVmax in 18F-FDG-PET is reportedly helpful for distinguishing tumor thrombosis from blood clot [6]. In this case, the tumor thrombus extended from the primary lesion to the root of the SMV (Fig. 2b), so we diagnosed it as a tumor thrombosis without PET-CT examination. 3D reconstruction of abdominal angiotomography showed the shape of the tumor thrombus, which extended from the ICV to the SMV over a distance of 3.3 cm, including the second jejunal vein (Fig. 2b, c).

Regarding the treatment of colorectal cancer accompanied by tumor thrombosis, surgical thrombectomy and radical resection of the primary tumor should be considered. Akabane et al. reported that right hemicolectomy and greater saphenous grafting were useful for avoiding massive bowel resection [7]. A procedure involving incising the vein, removing the tumor thrombus, and closing the vein with a prolene suture was reported by Sung et al. [8]. In our case, we decided to resect the SMV because the vein was full of tumor thrombus and the flow of blood inside the vein was not detected in the dynamic CT examination. We ligated the SMV with the tumor thrombus initially to avoid the movement of cancer fragments during surgical resection. At the resection of the SMV, we should pay particular attention to the intestinal blood flow. SMV thrombosis can cause intestinal infarction and bowel necrosis [9]. Recently, ICG fluorescence angiography with near-infrared (NIR) light has been introduced in the field of laparoscopic surgery. We can utilize this method for the real-time intraoperative assessment of intestinal artery supply [[10], [11], [12]]. After clamping the SMV for 30 min above the proximal side of the tumor thrombus, the small intestine showed no ischemic or congestive changes under the direct visualization, and good perfusion was confirmed using the NIR camera system after ICG injection (Fig. 4). The macroscopic intestinal finding was normal during the almost 8 h operation. Through this procedure, we could confirm the sufficient artery supply and vein return and could resect the SMV with tumor thrombus safely.

The prognosis of patients with venous tumor thrombosis originating from colorectal cancer may be poor considering the aggressive character. Otani et al. reported that, in the same meta-analysis, 10 of 41 cases with venous tumor thrombosis from colorectal cancer had liver metastatic recurrence after complete surgical resection of the tumor [2]. The mean survival time of those who had liver metastasis was 22.5 months. Akabane reported 12 cases of colon cancer accompanied by tumor thrombosis in the SMV and IMV. After surgical treatment, liver metastatic recurrence occurred in 4 of 12 cases [7]. However, following complete radical resection associated with adjuvant chemotherapy, some patients show long-term survival. Adjuvant chemotherapy was performed in 10 of 12 cases. The regimens and duration of adjuvant chemotherapy were various including FOLFOX, CapeOX, FOLFIRI, 5-FU/LV, TS-1 and so on. Of 10 cases that performed adjuvant chemotherapy, 3 cases survived for more than 2 years after the diagnosis. The effectiveness of adjuvant chemotherapy is still unclear. Adjuvant chemotherapy, even after complete surgical resection, may contribute to be a better prognosis considering micrometastasis, because venous tumor thrombosis increases the risk of liver metastatic recurrence. In this case, the patient did not receive adjuvant chemotherapy for his own will and he had a poor prognosis. If systemic chemotherapy was enabled, the prognosis might have been better. It is difficult to confirm the relationship between the prognosis and the recurrence of liver metastasis or the treatment procedure. But radical resection made oral ingestion possible and improved the quality of his life. A greater accumulation of evidence regarding the treatment procedure incidence of recurrence or metastasis, and survival time will help us determine the appropriate strategy for colorectal cancer accompanied with tumor thrombosis.

## 4 Conclusions

There is no absolute indication for surgical thrombectomy for colorectal cancer with venous tumor thrombus from the viewpoint of curative resection. A greater accumulation of evidence regarding the treatment, metastasis, and survival time of colorectal cancer will help us plan a strategy for patients with colorectal cancer accompanied by venous tumor thrombosis. Radical resection of the primary cancer combined with venous and adjuvant chemotherapy may be one of the treatment strategies. The surgical procedure of thrombectomy should be selected carefully considering bowel blood flow and oncological safety.

## Declaration of Competing Interest

There was no study sponsor and no funding provided for this project.

## Sources of funding

There was no study sponsor and no funding provided for this project.

## Ethical approval

This case report is exempt from ethical approval at our institution.

## Consent

Written informed consent was obtained from the patient’s family for publication of this case report and accompanying images. A copy of the written consent is available for review by the Editor-in-Chief of this journal on request.

## Author contribution

Yoshiaki Fujii wrote the manuscript. Yoshiaki Fujii, Kenji Kobayashi, Sho Kimura, and Shuhei Uehara performed the surgery and postoperative management. Shuji Takiguchi revised the manuscript.

## Registration of research studies

1. Name of the registry: Ascending colon cancer accompanied by tumor thrombosis in the superior mesenteric vein: A case report.
2. Unique identifying number or registration ID: researchregistry5520.
3. Hyperlink to your specific registration (must be publicly accessible and will be checked): https://www.researchregistry.com/browse-the-registry#home/registrationdetails/5e9dc2e0c1aaee00168cecfd/.

## Guarantor

YOSHIAKI FUJII.

## Provenance and peer review

Not commissioned, externally peer-reviewed.
